# Evaluation of Immunoregulatory Biomarkers on Plasma Small Extracellular Vesicles for Disease Progression and Early Therapeutic Response in Head and Neck Cancer

**DOI:** 10.3390/cells11050902

**Published:** 2022-03-05

**Authors:** Jadwiga Jablonska, Malwina Rist, Ilona Spyra, Luisa Tengler, Maksim Domnich, Benjamin Kansy, Bernd Giebel, Basant Kumar Thakur, Nicole Rotter, Stephan Lang, Sonja Ludwig

**Affiliations:** 1 Department of Otorhinolaryngology, Head and Neck Surgery, University Hospital Essen, University of Duisburg-Essen, Hufelandstr. 55, 45147 Essen, Germany; jadwiga.jablonska@uk-essen.de (J.J.); malwina.rist@uk-essen.de (M.R.); ilona.spyra@uk-essen.de (I.S.); maksim.domnich@uk-essen.de (M.D.); benjamin.kansy@uk-essen.de (B.K.); stephan.lang@uk-essen.de (S.L.); 2 German Cancer Consortium (DKTK) Partner Site Düsseldorf/Essen, 45147 Essen, Germany; 3 Department of Otorhinolaryngology, Head and Neck Surgery, University Hospital Mannheim, Medical Faculty Mannheim, University of Heidelberg, Theodor-Kutzer-Ufer 1-3, 68167 Mannheim, Germany; luisa.tengler@medma.uni-heidelberg.de (L.T.); nicole.rotter@umm.de (N.R.); 4 Institute for Transfusion Medicine, University Hospital Essen, University of Duisburg-Essen, Hufelandstr. 55, 45147 Essen, Germany; bernd.giebel@uk-essen.de; 5 Department of Pediatrics III, Pediatric Hematology & Oncology, University Hospital Essen, University of Duisburg-Essen, 45147 Essen, Germany; basant-kumar.thakur@uk-essen.de

**Keywords:** exosomes, small extracellular vesicles (sEVs), immunoregulation, biomarkers, therapy response

## Abstract

Head and Neck Cancers (HNCs) have highly immunosuppressive properties. Small extracellular vesicles (sEVs), including exosomes, nanosized mediators of intercellular communication in the blood, carry immunosuppressive proteins and effectively inhibit anti-tumor immune responses in HNCs. This study evaluates immunosuppressive markers on sEVs from 40 HNC patients at different disease stages and 3- and 6-month follow-up after surgery and/or chemoradiotherapy. As controls, sEVs from normal donors (NDs) are examined. Immunoregulatory surface markers on sEVs were detected as relative fluorescence intensity (RFI) using on-bead flow cytometry, and their expression levels were monitored in the early and late stages of HNC and during follow-up. In parallel, the sEV-mediated apoptosis of CD8^+^ Jurkat cells was assessed. Together with TGF-β1 and PD-L1 abundance, total sEV proteins are elevated with disease progression. In contrast, total sEV protein, including TGF-β1, PD-1 and PD-L1, decrease upon therapy response during follow-up. Overall survival analysis implies that high sEV PD-1/PD-L1 content is an unfavorable prognostic marker in HNC. Consistently, the sEV-mediated induction of apoptosis in CD8^+^ T cells correlates with the disease activity and therapy response. These findings indicate that a combination of immunoregulatory marker profiles should be preferred over a single marker to monitor disease progression and therapy response in HNC.

## 1. Introduction

Head and neck cancer (HNC) is one of the most common cancer entities worldwide with an approximate incidence of 800,000 new cases [1]. Due to their localization, most HNCs are already advanced at diagnosis, resulting in failed therapy response and tumor progression despite multimodal treatment regimens [2]. To date, multimodal therapeutic approaches of HNCs consist of surgery plus adjuvant (chemo)radiotherapy (CRT) or primary CRT alone. Additionally, novel therapeutic strategies, such as immunotherapies, stayed beyond the expectations, resulting only in ca. 15% response to the treatment [3]. One major cause for this phenomenon could be the relatively low expression of immune-checkpoint molecules, i.e., PD-1, in HNCs that are the targets of such therapies [3]. At the same time, HNC is characterized by profound immune suppression. Therefore, diagnostic, minimal-invasive tools and biomarkers are necessary to detect this cancer at the early stage and to monitor disease progression. Adjustments of therapeutic regimens at early would significantly improve patient prognosis.

Small extracellular vesicles (sEVs), including exosomes, have recently been recognized as potential diagnostic tools for cancer progression and have become emerging key players in tumor immunology. sEVs are nano-scaled lipid bilayer vesicles that are released from almost all living cells into their environment and mediate intercellular communication [4,5]. sEVs are composed of the nucleic acid, lipid and protein cargo of their cells of origin. Mainly, tumor cells from HNC release high amounts of sEVs (tumor-derived exosomes, TEX), which can exert immunosuppressive functions and promote immune evasion due to their protein content [6,7,8]. These functional characteristics distinguish exosomes from other extracellular vesicles (microvesicles or apoptotic bodies) in the blood [9]. In the past, size-exclusion chromatography as the best isolation method to retrieve undamaged and functionally active sEVs has been optimized by us and others [10,11,12]. Additionally, we have previously demonstrated that some sEV features, such as the protein content or immunosuppression of T cells, are dependent on the disease activity of HNCs [8]. Hence, there are multiple implications that sEVs can be used as early indicators for response to therapy [13]. However, the exact correlation between the disease progress and sEV composition must be elucidated. In this study, we evaluate the role of plasma-derived sEVs from HNC patients as biomarkers to estimate disease and therapy prognosis.

## 2. Materials and Methods

### 2.1. Plasma Specimens

Venous blood specimens were collected from head and neck cancer patients (HNC, n = 40) attending the Department of Otorhinolaryngology, Head and Neck Surgery of the University Hospital Essen from 2016 to 2019 and from age- and gender-matched normal donors (ND, n = 13). All study participants signed an informed consent before study inclusion. Although most patients appeared to follow-up, some refused blood withdrawal, which is why only one patient was studied with a recurrent disease and had to be excluded in the follow-up analysis. The local ethics committee approved the study (#16-7135-BO). For plasma separation, the blood samples were centrifuged at 1000× *g* for 10 min at room temperature (RT) and frozen at −80 °C in aliquots for storage.

### 2.2. sEV Preparation from Plasma by Size-Exclusion Chromatography

sEVs from plasma were prepared by size exclusion chromatography as previously described by Hong et al. [10]. Briefly, freshly thawed plasma specimens were differentially centrifuged at 2000× *g* for 10 min at RT and 14,000× *g* for 30 min at 4 °C, followed by ultrafiltration (Millipore filter, 0.22 μm, Merck Millipore, Burlington, MA, USA). Self-made mini-size-exclusion chromatography columns with 10 mL Sepharose 2B gel volume (GE Healthcare, Chicago, IL, USA, cat. GE17-0140-01) were prepared and 1 mL plasma was loaded and eluted with phosphate-buffered saline (PBS) to retrieve 1 mL fractions. The 4th fraction was collected and used for further studies.

### 2.3. Transmission Electron Microscopy (TEM)

TEM of sEVs from NDs and HNC patients was performed at the Electron Microscopy Core Facility of Heidelberg University. Glow-discharged carbon-coated formvar grids (75 mesh copper; 3 nm carbon on formvar) were placed on a 20 μL drop of freshly prepared sEV fraction, for negative staining. The samples were allowed to adsorb to the carbon for ~10 s, washed three times briefly on a drop of double distilled water, stained on 2 drops of 3% *w*/*v* aqueous uranyl acetate, blotted with filter paper and dried. Micrographs were recorded using a JEM1400 transmission electron microscope (JEOL Ltd., Akishima, Tokyo, Japan) with a bottom-mounted 4K CMOS camera and a lens magnification of 25,000× (TemCam F416; TVIPS, Gauting, Germany).

### 2.4. BCA Protein Assay

According to the manufacturer’s instructions, the protein content of the obtained plasma sEV preparations was analyzed using Pierce^TM^ BCA protein assay (Thermo Fisher Scientific, Waltham, MA, USA). The total protein of sEVs was determined directly after sEV preparation by size-exclusion chromatography (unconcentrated samples). For Western blots or bead-bound flow cytometry, sEVs were concentrated on centrifugal filter units (100 kDa, Merck Millipore, Burlington, MA, USA) and the protein amount was detected by BCA.

### 2.5. Western Blots

Samples were mixed with Laemmli sample buffer with or without DTT (non-reducing conditions were applied for CD63 and CD81) and denatured for 5 min at 90 °C. Afterwards, 10 μg of exosomes were separated by electrophoresis on 10% SDS-polyacrylamide gel and transferred onto a nitrocellulose membrane (Thermo Fisher Scientific, cat. 88018). The membrane blocking was performed for 2 h in 5% skim milk and 0.1% Tween20 in PBS (for anti-CD9, anti-CD63, anti-TSG101) or 5% BSA and 0.1% Tween in PBS (for anti-CD81). Incubation with the primary antibodies, anti-CD63 (Invitrogen, Waltham, MA, USA, cat. 10628D, 1:1500), anti-CD9 (Santa Cruz Biotechnology, Dallas, TX, USA, cat. Sc-13118, 1:500), anti-CD81 (Biorbyt, Cambridge, U.K., cat. Orb388959, 1:500), anti-TSG101 (Becton Dickinson, Franklin Lakes, NJ, USA, cat. 612697, 1:800), was performed overnight at 4 °C. After washing, HRP-conjugated secondary antibody (IgG goat-anti-mouse, Dianova, Hamburg, Germany, cat. 115-035-003, 1:10,000) was added and incubated for 1 h at RT. According to the manufacturer’s instructions, the chemiluminescent signal was elicited by AceGlow™ Chemiluminescence Substrate (VWR Life Science, Radnor, PA, USA, cat. 730-1511).

### 2.6. Nanoparticle Tracking Analysis (NTA)

Nanoparticle tracking analysis (NTA) was performed on ZetaView (Particle Metrix, Inning am Ammersee, Germany) to determine the size distribution and concentration of the isolated particles. Briefly, freshly prepared plasma sEV samples from HNC patients were diluted at 1:150 to 1:4000 in PBS and from ND at 1:100 to 1:1000 in PBS and measured at eleven test ranges with five cycles at 4 °C, a sensitivity of 92.0 and a shutter of 70. As controls, polystyrene beads of 100 nm in size and PBS were recorded. The concentration and size ranges were calculated by NTA 2.0 analytical software (Particle Metrix, Inning am Ammersee, Germany).

### 2.7. Flow Cytometry of Immunosuppressive Markers on sEVs

To detect surface markers on exosomes, CD63-coupled magnetic streptavidin beads were used to bind and examine sEVs, as previously described by Theodoraki et al. [14,15]. sEVs were concentrated on centrifugal filter units (Merck Millipore, Burlington, MA, USA) to reach a 10 μg protein/100 μL PBS concentration. The concentrated sEVs were co-incubated with biotinylated anti-CD63 mAb (BioLegend, San Diego, CA, USA, cat. 353018, 1:50) for 2 h at RT. Next, magnetic ExoCap streptavidin beads (MBL International, Woburn, MA, USA) were co-incubated in the anti-CD63-cocktail for 2 h at RT. The bead-bound sEVs were washed once with washing buffer from the kit and blocked in heat-inactivated mouse serum (Invitrogen, Waltham, MA, USA, cat. 10410, 1:5) for 30 min and diluted in 100 μL PBS.

Subsequently, Abs and matching isotype controls were used: anti-PD-1 FITC (cat. 367311, 1:20), anti-PD-L1 PE (cat. 393607, 1:20), anti-TGF-β1 APC (cat. 349705, 1:20), anti-CD44 FITC (cat. 338803, 1:20), anti-FasL PE (cat. 306406, 1:50), anti-Fas APC (cat. 305611, 1:20). All antibodies and isotypes were purchased from BioLegend (San Diego, CA, USA). Abs and corresponding isotypes were co-incubated with the sEV/anti-CD63Ab/bead-complex for 1 h at RT. Briefly, samples were washed two times and resuspended in 200 μL PBS. Antigen detection was performed using a FACS Canto II flow cytometer (BD Bioscience, Franklin Lakes, NJ, USA) and analyzed using the FACSDiva Software 8.0 (BD Bioscience). Results are shown as relative fluorescence intensity (RFI), calculated by dividing the mean fluorescence intensity (MFI) of a specific antibody staining by the MFI of the matching isotype control (RFI = MFI (AB)/MFI (ISO)).

### 2.8. sEV-Mediated Apoptosis Induction in CD8^+^ Jurkat Cells

To assess the apoptosis induction, CD8^+^ Jurkat cells were used as a model cell line as described previously [8]. CD8^+^ Jurkat cells were cultivated in RPMI medium (Gibco, Thermo Fisher Scientific, Waltham, MA, USA) supplemented with 10% microvesicle-depleted fetal bovine serum (FBS, Gibco) and 1% penicillin/streptomycin (Gibco) at standard conditions (37 °C, 5% CO_2_). A purity of over 95% of CD8 positive cells was confirmed before experiments. CD8^+^ Jurkat cells were pre-plated at a 10^6^ cells/mL concentration in a 96-well-plate (10^5^ cells/well). After 24 h, 50 μL freshly prepared sEVs (2–3 μg) or as control, 50 μL PBS, were co-incubated for another 24 h at 37 °C. On day 3, CD8^+^ cells were stained using PE-conjugated Annexin V (BD Bioscience, Franklin Lakes, NJ, USA) was performed according to the manufacturer’s instructions. As a positive control, dead cells (boiling–freezing–thawing procedure) were used. All samples were measured using FACS Canto II flow cytometer (BD Bioscience, Franklin Lakes, NJ, USA) and analyzed using BD FACSDiva Software 8.0 (BD Bioscience).

### 2.9. Statistical Analysis

Statistical analysis was performed using GraphPad Prism version 8. Scatter plots represent means and standard deviation (SD). The bar in the box plots shows the median of the values. The box displays the interquartile range and the whiskers extend the interquartile range, including outlier values. For parametric data, unpaired t-tests were used, while non-parametric data were analyzed using the Mann–Whitney U test. Overall survival (OS) was determined as the time from tumor resection to the date of cancer-related death (event) by Kaplan–Meier analysis. All patients that did not reach this event were censored (tumor-unrelated death). To estimate the differences between the groups log-rank tests were performed. A *p*-value below 0.05 was considered significant.

## 3. Results

### 3.1. Clinicopathological Characterization of the Study Participants

To evaluate the possible role of sEVs as biomarkers for HNC progression, we first characterized their content and functionality. To this end, we analyzed 40 HNC patients at different tumor stages and 13 normal controls (ND).

The clinicopathological characteristics of the study participants are summarized in Table 1. Study participants, exclusively HNC patients, were predominantly male (73%), with a mean age of 64 years, ranging between 32–85 years. Primary tumor locations were in decreasing order: larynx (40%), oropharynx (33%), oral cavity (23%) and hypopharynx (5%). The majority of the oropharynx patients were HPV-negative according to the clinical surrogate marker p16 (77%). As risk factors are concerned, most patients had consumed tobacco (80%) and/or alcohol (55%) at tumor diagnosis or in the past. At diagnosis, most patients exhibited rather small tumors (T1/2 = 63%), no spread to nearby lymph nodes (N0 = 53%) and/or no distant metastasis (M0 = 100%). According to the 8th edition of the UICC classification, more HNC patients suffered from advanced tumor stages (UICC III/IV = 55%) than early tumor stages (UICC I/II = 45%). HNC tumors were moderately differentiated (G2 = 70%). As tumor treatment, patients received either surgery alone (30%), a combination of surgery plus adjuvant (chemo)radiotherapy (60%) or primary chemoradiotherapy (10%). A total of 30 HNC patients presented no evident disease (NED) during follow-up, while 4 patients suffered from tumor progression and died. Three patients (10%) did not appear at their follow-up appointments. One patient suffered from local recurrence and perished in the follow-up period.

### 3.2. Morphology, Size Distribution, Concentration and Protein Content of Plasma sEVs

**Figure 1:**
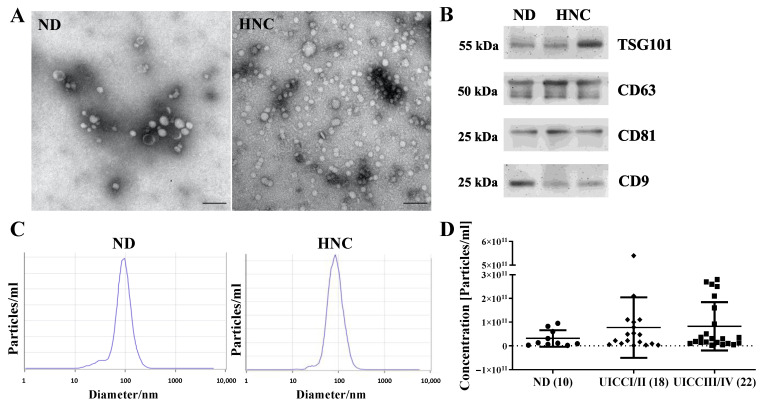
Characterization of sEVs from ND and HNC by morphology, protein content and size. (**A**) Representative TEM pictures from ND and HNC show the typical vesicular shape and size ranges of sEVs. The black scale bar represents 100 nm. (**B**) Western blots show sEV-specific antigens (tetraspanins and TSG101) for one ND (normal donor) and two HNC patients. (**C**) Size distribution and particle concentration of the prepared sEVs were measured by NTA. Representative pictures of the size distribution of sEVs from one ND and one HNC patient are shown. (**D**) The particle concentration is elevated in HNC patients compared to NDs (*p* > 0.05).

sEVs were prepared from HNC patients and normal donors (ND) by size exclusion chromatography and were characterized by transmission electron microscopy (TEM) and Western blots. Microscopy confirms the presence of sEVs as morphologically intact vesicles with sizes ranging between 40 and 100 nm. No significant morphological differences could be observed between NDs and HNC patients (Figure 1A).

sEVs were concentrated on Millipore filters (100,000 MWCO) to load 10 μg per Western blot lane and examined for classical sEV markers, according to MISEV 2018 [9]. As shown in Figure 1B, the typical EV markers, such as tetraspanins (CD63, CD9, CD81) and TSG101, are present in our sEV preparations.

To determine the size range and particle concentration of the prepared sEVs, the samples were analyzed using NTA. The size range was consistent in all patients, ranging between 80 and 130 nm, with a mean diameter of 99 nm (Figure 1C). The particle concentration was elevated in HNC patients compared to ND. However, no significant difference was observed for different tumor stages (early vs. advanced stage) (Figure 1D).

**Figure 2:**
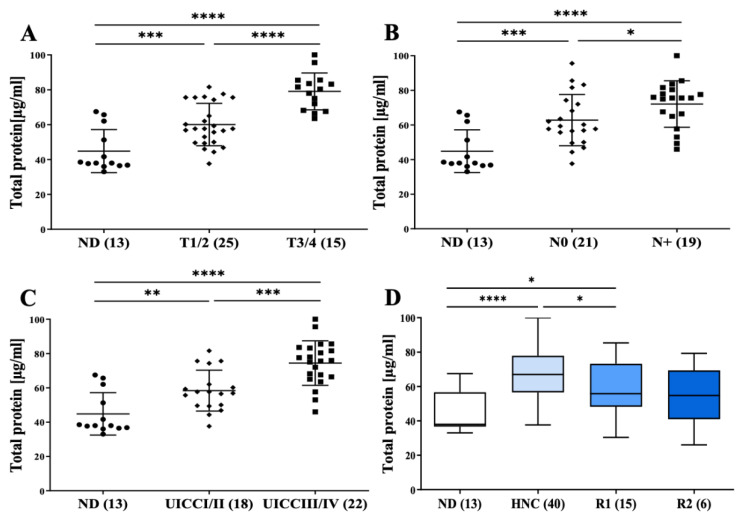
The total protein content of plasma sEV preparations as an indicator of progressed disease and therapy response. The total sEV protein content (of unconcentrated samples) is significantly higher in HNC patients than NDs and increases with (**A**) bigger tumor size, (**B**) positive nodal status and (**C**) advanced tumor stage. (**D**) The decrease in protein content indicates response to therapy. ND: normal donor; HNC: HNC patient; R1: patients after 3 months-follow-up; R2: patients after 6 months follow-up. *p* < 0.05 was considered significant. (* *p* < 0.05; ** *p* < 0.01, *** *p* < 0.001, **** *p* < 0.0001).

After the preparation on mini-SEC, the sEV protein content was determined by BCA protein assays. Interestingly, while protein content of sEVs prepared from ND was low, sEV preparations from HNC patients showed significantly increased protein concentration along with disease progression: higher in early stages I/II and further increased in advanced stages III/IV (*p* < 0.01; Figure 2A–C). Of note, a total EV protein content decreased after treatment, which could be an early indicator for response to therapy (*p* < 0.05; Figure 2D).

### 3.3. Immunomodulatory Surface Markers on sEVs from HNC Patients with Different Tumor Stages

**Figure 3:**
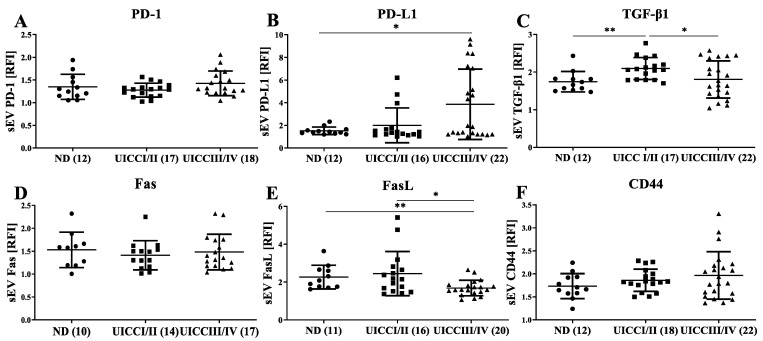
Altered expression of sEV immunosuppressive markers during tumor progression. CD63-coated streptavidin beads were used to capture sEVs from HNC patients and NDs. Captured sEVs were stained for various immunosuppressive markers. (**A**,**D**,**F**) PD-1, Fas and CD44 show similar expression on sEVs from HNC patients and NDs. (**B**) PD-L1 expression increases with tumor stages/tumor progression (UICC III/IV > I/II). (**C**) TGF-β1 is significantly overexpressed in early HNC stages (UICC I/II). (**E**) The FasL expression on exosomes decreases along with HNC tumor progression (UICC III/IV < I/II). A *p*-value below 0.05 was considered as significant (* *p* < 0.05, ** *p* < 0.01).

As sEVs are suggested to play a role in immunomodulation during cancer progression, we assessed the expression of immunomodulatory molecules on their surface [8]. Freshly isolated sEVs were captured by CD63-coated streptavidin beads as described previously by Theodoraki et al. [7]. The expression of immunomodulatory cell surface markers, such as PD-1, PD-L1, TGF-β1, Fas, FasL and CD44, was assessed using flow cytometry. Interestingly, while PD-1, Fas and CD44 presented comparable expression levels on captured sEVs between ND and HNC patients (Figure 3A,D,F), PD-L1 expression on sEVs significantly increased during HNC progression (*p* < 0.05; Figure 3B). TGF-β1 levels on sEVs were significantly elevated in early tumor stages I/II compared to ND, but reduced at advanced tumor stages III/IV (*p* < 0.01; Figure 3C). Conversely, FasL expression on sEVs is the highest in ND, is decreased in early tumor stages I/II and the lowest in advanced tumor stages III/IV (*p* < 0.05, Figure 3E).

### 3.4. Modulation of Immunosuppressive Markers on sEVs of HNC Patients following Treatment

**Figure 4:**
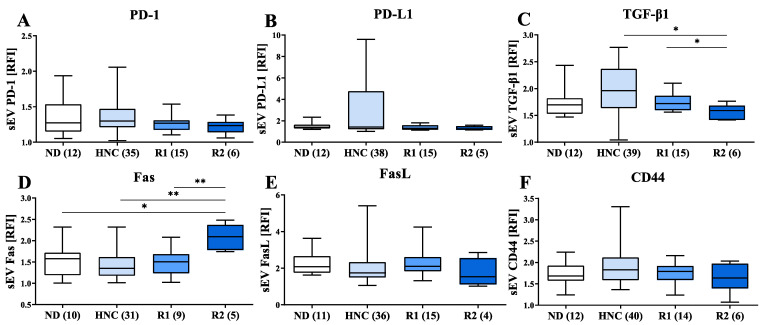
Expression of immunosuppressive markers on sEVs during follow-up. sEVs were captured on CD63-coated streptavidin beads and stained for immunosuppressive proteins on sEVs prepared from HNC patients and during follow-up period: after 3 (R1) and 6 (R2) months after tumor therapy. (**A**–**C**) PD-1, PD-L1 and TGF-β1 expression levels on plasma sEVs decrease during follow-up. (**D**) Fas expression is upregulated at 6 months follow-up. (**E**,**F**) FasL and CD44 expression on sEVs are stable during follow-up. *p* values below 0.05 were considered significant (* *p* < 0.05, ** *p* < 0.01).

To assess changes in the expression of immunosuppressive markers on sEVs isolated from HNC patients after tumor treatment, the presence of PD-1, PD-L1 and TGF-β1 was evaluated during patient control examination, three (R1) and six (R2) months after completion of tumor therapy (R = Recall). Without reaching statistical significance, declined levels of PD-1 and PD-L1 were observed within post-tumor therapy sEV samples (Figure 4A,B). Similarly, the abundance of TGF-β1 in sEV samples was significantly lower following anti-tumor therapy than in samples before treatment (Figure 4C). Fas abundance is significantly higher during the initial 6 months of follow-up (up to R2, Figure 4D), and decreased during further follow-up. FasL and CD44 abundances on the sEVs of HNC patients are not significantly altered in the follow-up sEV samples (Figure 4E,F).

### 3.5. sEVs from HNC Patients Induce the Apoptosis of CD8^+^ T Cells

**Figure 5:**
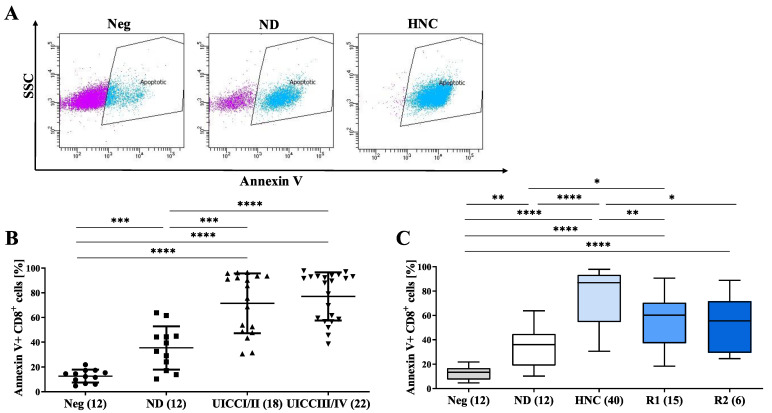
The induction of apoptosis of CD8^+^ Jurkat cells depends on HNC disease activity. (**A**) Representative pictures of the gating strategy of sEV-mediated apoptosis in CD8^+^ Jurkat cells. (**B**) sEVs from HNC patients induce significantly more apoptosis in CD8^+^ Jurkat cells than sEVs from ND. (**C**) The apoptosis induction of CD8^+^ cells decrease during follow-up and almost equalizes NDs at six months follow-up. Neg: Negative control (PBS/no sEVs). A *p*-value below 0.05 was considered as significant (* *p* < 0.05; ** *p* < 0.01; *** *p* < 0.001; **** *p* < 0.0001).

Since we observed a stage-dependent regulation of molecules involved in the regulation of apoptosis, such as TGF-β, PD-L1 and PD-L1 on sEVs during follow-up, we assessed the pro-apoptotic ability of sEVs prepared from HNC patients at the different cancer stages. To this end, we incubated sEVs prepared from HNC patients at different disease stages and from normal donors with CD8^+^ Jurkat cells and assessed their apoptosis (gating strategy Figure 5A). We observed that sEVs prepared from HNC patients show significantly higher apoptotic activity than sEVs from healthy individuals (ND) (Figure 5B). Interestingly, the pro-apoptotic activity of sEVs does not vary between cancer stages, but shows a significant decrease in recovered patients (Figure 5C).

These results suggest an essential role of sEVs in inducing CD8^+^ T-cell apoptosis, thus influencing anti-tumor immunity. The presence of pro-apoptotic molecules on sEVs obtained from HNC patients points towards tumor progression and could be used as a useful biomarker.

### 3.6. Five-Year Overall Survival (OS) of HNC Patients

**Figure 6:**
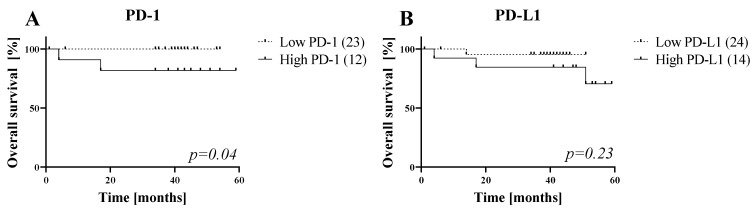
Overall survival of HNC patients depends on sEV PD-1 and PD-L1 abundance. (**A**) Higher sEV PD-1 and (**B**) PD-L1 levels before therapy indicate worse survival rates in Kaplan–Meier analysis. Log-rank test revealed a significantly worse prognosis for patients with elevated PD-1 values on sEVs (lower right corner, *p* = 0.04).

To analyze the prognostic relevance of the immunosuppressive markers on sEVs, we performed Kaplan–Meier analysis for all markers before any therapy (Figure 6 and Supplementary Figure S1). Overall survival (OS) rates of HNC patients with higher sEV PD-1 abundance show significantly worse prognosis than patients with low sEV PD-1 abundance (log-rank test, *p* = 0.04). Similar observations were made for sEV-associated PD-L1, where high PD-L1 sEV level worsens OS (log-rank test, *p* > 0.05). Interestingly, no other immunosuppressive markers show significant prognostic value in our patient cohort (Supplementary Figure S1).

## 4. Discussion

Previously, we reported that sEVs from HNC patients carry immunosuppressive protein cargo and mediate suppressive effector T-cell functions [8]. While immunosuppressive markers, such as PD-1/PD-L1, were more prevalent in patients with active disease than in NED following therapy or NDs, it remained unclear whether they vary in the course of disease progression or could indicate tumor recovery. In this paper, we set up to clarify this issue. We compared immunosuppressive proteins on sEVs from plasma of HNC patients and normal donors by on-bead flow cytometry and evaluated the sEV-mediated apoptosis induction of CD8^+^ lymphocytes during the first six months of follow-up.

As proof of concept, we demonstrated that total sEV protein content and the capacity to induce CD8^+^ lymphocyte apoptosis by sEVs correlate with the disease activity. This agrees with the data shown by us and others before [7,8]. Additionally, the observed decrease in total sEV protein content indicated the positive therapy response after a 3-month follow-up. This is based on the assumption that the relative tumor sEV-proportion in the blood of HNC patients increases in active disease and decreases after successful therapy [16]. CD44 and TGF-β have been considered essential markers for cell proliferation and cancer progression for many years [17,18]. However, our results indicate that CD44 neither changes upon cancer progression nor in NED patients during the follow-up. Interestingly, the isoform variant CD44v3 has been recently described to regulate epithelial-mesenchymal transition (EMT) and promote metastases in breast cancer [19,20]. In HNCs, CD44v3 overexpression correlates with a higher migratory potential, proliferation and resistance to chemotherapy, and thus a poorer prognosis for the patient [21,22]. Consequently, CD44v3 may be a better biomarker in tumor tissue and on tumor sEVs isolated from a liquid biopsy [16,23] and should be preferred over CD44 in future studies on HNCs.

The overexpression of TGF-β1 has been reported to promote proliferation, angiogenesis and metastases in HNCs [24,25]. Our experiments show that, in early stage HNCs, sEV TGF-β1 content increases and decreases after therapy. Interestingly, TGF-β1 is known to induce PD-L1 expression on tumor-derived sEVs and thus effectively suppresses anti-cancer CD8 T-cell immune responses [26]. In agreement with this, we demonstrated that the PD-1/PD-L1 axis is elevated on tumor sEVs upon disease progression and reduced after response to therapy. PD-L1 is an immunosuppressive ligand associated with immune evasion in cancer [27]. Multiple studies demonstrated elevated sEV PD-L1 levels in different cancer entities, which correlated with poor patient prognosis [28,29]. Moreover, Theodoraki et al. observed that low sEV PD-L1 content is linked to shorter disease-free survival in HNC cancer and, thus, an unfavorable prognostic factor [13]. On the contrary, we observed a prolonged OS in patients with low sEV PD-1 and PD-L1 levels (although not significantly) before therapy. The prognostic value of elevated PD-1/PD-L1 in HNCs has been controversially discussed as either a bad [30] or good prognostic factor [31,32,33,34]. High sEV PD-L1 has been correlated with therapy resistance in HNCs [7,13] and other tumors, such as melanoma [35]. sEV PD-L1 is upregulated by IFN-γ expression, commonly present in the tissue microenvironment and induces apoptosis upon interaction with PD-1 on CD8^+^ lymphocytes [35]. This suggests the dual role of the PD-1/PD-L1 axis, not only being a sign of immune evasion, but also of immune activation.

Although the PD-L1/PD-1 axis seems to be the most promising biomarker of patient survival, PD-L1 is not expressed by all HNC patients [31,32]. Moreover, as we mentioned previously, its relevance for patient survival is controversial. Therefore, a decision exclusively based on this marker might not be sufficient in HNCs. Interestingly, we could identify another maker on sEVs that seems to correlate with better patient prognosis. sEV-associated FasL levels are reduced in advanced HNCs and sEV Fas is increased in NED patients up to six months after therapy. We have previously shown that sEVs from HNC patients with active disease express Fas and FasL. While Fas expression decreases, FasL remains consistent in NED patients [8]. Other studies demonstrated that elevated Fas/FasL expression is a positive prognostic marker in oral and laryngeal cancer [36,37], and also various other cancers (gastric: [38], colorectal: [39], esophageal: [40]). The sEV Fas/FasL expression might reflect the expression patterns of the cells of origin. Immunosuppression mediated by the Fas/FasL pathway is considered crucial for cancer-mediated immunosuppression [41] and the interaction of FasL on sEVs with the Fas receptor on T cells induces apoptosis in CD8^+^ T cells [42]. In small cell lung cancer and breast cancer, the upregulation of Fas on tumor cells reflects a higher susceptibility of cancer cells to platinum-based therapies [43,44]. The loss of FasL expression on sEVs in advanced disease might represent the downregulation of the apoptosis signaling pathways by the tumor cells as a matter of tumor resistance and the upregulation of Fas suggests the recovering immune system after successful therapy.

While our study investigated potential biomarkers on sEVs, which impair anti-tumor immune responses, we identified the following limitations. The heterogeneity of HNCs is a very well-recognized phenomenon, which is based on their different etiology (i.e., noxious agents, HPV and EBV) and localizations within the upper aerodigestive tract. Our study investigated a mixed patient cohort representative for the heterogeneity of HNCs to unravel biomarkers that are not limited to a certain subsites or etiologies. Due to time constrains and low participation rates during follow-up, the study was performed in a relatively small cohort of patients. These factors could impair the outcome of our study. However, in spite of the low number of patients and NED individuals, we observed various potential markers on sEVs and could identify relatively little variety in our results. Therefore, we are convinced that the significance of the obtained results is provided. Possibly, the increase in the examined cohort would lead to an elevated statistical significance of our results, but, based on our observations, we do not expect any changes in the direction of our observations.

## 5. Conclusions

Various immunotherapies have been established to treat patients with advanced, recurrent HNCs. While they are effective in some cancer entities, such as melanoma, non-small cell lung cancer, colorectal cancer or breast cancer [45,46,47], immunotherapies in HNC patients are disappointing, with only up to 15% of patients responding to the treatment [3]. The role of sEVs in this process and their interference with the immune system and immunotherapies are an unsolved issue. Clearly, the immunosuppressive marker on sEVs serves as valuable biomarkers, and affects established and novel therapy regimens [48]. Considering the variability of marker expression on sEVs in HNCs, a combined panel of multiple biomarker expression should be preferred over a single protein marker in patients. Further biomarker studies are necessary to examine the potential and predictive value of marker combinations and therefore should be continued in the future.

## Figures and Tables

**Table 1:** Clinicopathological characteristics of the HNC patients (n = 40).

	N	%
**Age (mean** **±** **SD; years)**		
≤63.7 ± 9.1	18	45.0
>63.7 ± 9.1	22	55.0
**Gender**		
Male	29	72.5
Female	11	27.5
**Tumor site**		
Oral cavity	9	22.5
Oropharynx	13	32.5
Larynx	16	40.0
Hypopharynx	2	5.0
**HPV status ^1^**		
Negative	10	76.9
Positive	3	23.1
**Smoking**		
Never	8	20.0
Former	6	15.0
Current	26	65.0
**Alcohol consumption**		
None	18	45.0
Former	7	18.5
Current	15	37.5
**Tumor size**		
T_1_	10	25.0
T_2_	15	37.5
T_3_	11	27.5
T_4_	4	10.0
**Lymph node status**		
N_0_	21	52.5
N_+_	19	47.5
**Metastases**		
M_0_	40	100.0
**UICC stages ^2^**		
I	8	20.0
II	10	25.0
III	9	22.5
IV	13	32.5
**Grading**		
G_1_	1	2.5
G_2_	28	70.0
G_3_	10	25.0
Missing	1	2.5
**Therapy**		
Surgery	12	30.0
Surgery plus CRT	24	60.0
Primary CRT	4	10.0
**Disease course after therapy**		
No evident disease (NED)	30	75
Local recurrence	2	5
Distant metastasis	1	2.5
Second carcinoma	1	2.5
Deceased	4	10.0
Unknown/Loss of follow-up	3	7.5

n: number of patients; SD: standard deviation; CRT: chemoradiotherapy; NED: no evident disease; ^1^ HPV status was determined by p16 immunohistochemistry for all patients with oropharynx carcinoma; ^2^ According to the UICC TNM classification, 8th edition.

## Data Availability

Supplementary data were uploaded as Supplementary Materials.

## Supplementary Materials

The following supporting information can be downloaded at: https://www.mdpi.com/article/10.3390/cells11050902/s1. Figure S1: Overall survival analysis of HNC patients for the immunoregulatory markers Fas, FasL, TGF-β1, and CD44.
