# Supplementary material for: Evaluation of Immunoregulatory Biomarkers on Plasma Small Extracellular Vesicles for Disease Progression and Early Therapeutic Response in Head and Neck Cancer
Source: Cells. 2022 Mar 5;11(5):902. doi: 10.3390/cells11050902 (PMC8909035; doi:10.3390/cells11050902)
Supplement: Supplementary file 1 [file cells-11-00902-s001.zip › cells-1614473-supplementary.pdf]

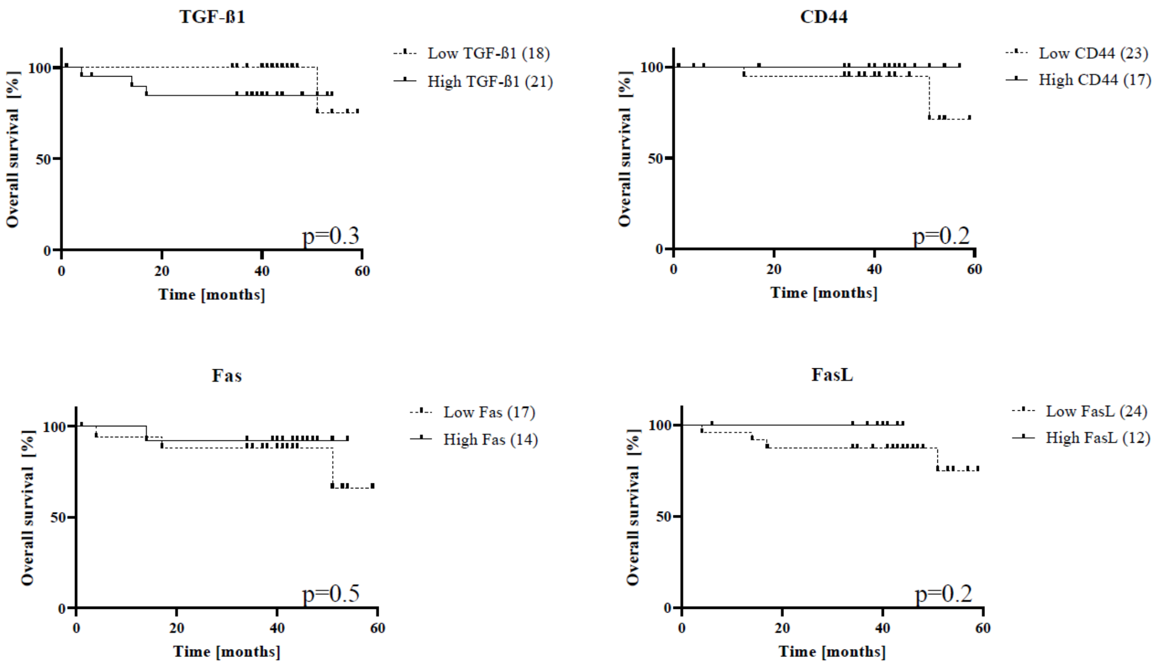

Supplementary Figure S1: Overall survival analysis of HNC patients for the immunoregulatory markers Fas, FasL, TGF-β1, and CD44.
